# Treating neglected tropical diseases

**Published:** 2013

**Authors:** Adrian Hopkins

**Affiliations:** Director: Mectizan Donation Program, Georgia, USA.

**Figure:**
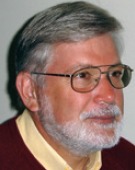
Adrian Hopkins

The name neglected tropical diseases (NTDs) covers a range of diseases that cause disability, early death, and slowed physical and mental development. The first two in entries Table 1 are diseases that cause blindness. These diseases of neglected and impoverished peoples maintain a cycle of poverty and delayed development of the populations affected. The diseases themselves have been neglected in the push to control malaria, TB and AIDS.

The NTDs fall into two main groups. The first group, which we will deal with here, are those for which we have tools (easy community diagnosis or mapping, as well as the drugs or medicines). These NTDs can be treated – where safe to do so – in large populations of patients using a mass drug administration (MDA) strategy. Dosages are standardised, and a dose pole can be used to measure the height of a person in order to calculate the dose required.

The second group of diseases, which we will not discuss here, require either more difficult or costly diagnosis and the people affected often need individualised treatment.

Treatment with drugs is important. Repeated annual or semi-annual drug distribution can lower the prevalence of a disease and, in some settings, eliminate transmission. However, in order to maintain these gains, more intensive efforts need to be made to provide safe water, sanitation, and hygiene. Specific measures are also required in trachoma and lymphatic filariasis (LF) to address the symptoms and consequences of these diseases, e.g. trichiasis surgery to correct in-turned eyelashes and prevent corneal scarring in people affected by trachoma and hydrocoele surgery for male genital deformity due to LF. The existence of an MDA programme is not a reason to ignore these other measures.

**Figure:**
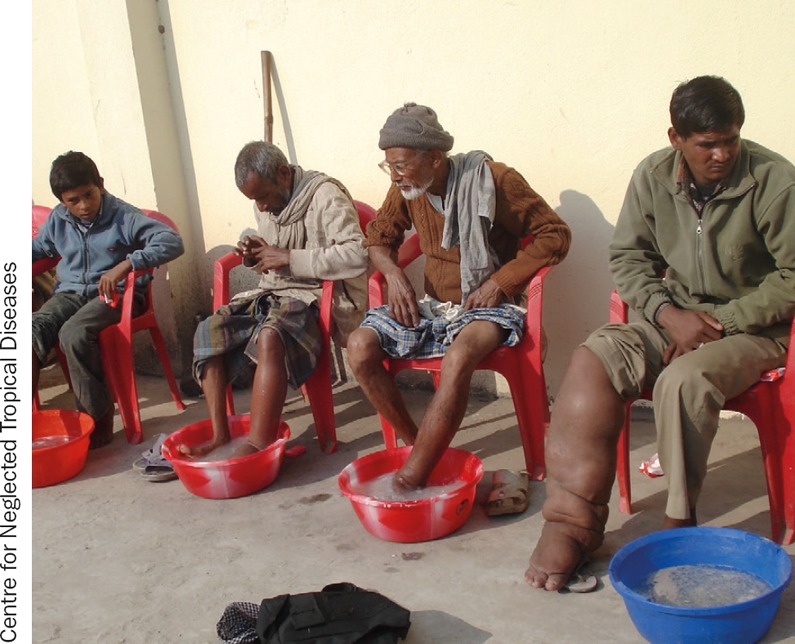
Lymphatic filariasis patients learn about the benefits of foot washing. SRI LANKA

**Table 1.** Current treatment guidelines for the five neglected tropical diseases for which mass drug administration is possible

Disease	Drugs and Dosages	Threshold for implementation	Frequency of Intervention
**Trachoma**	Azithromycin 20mg/kg, with a maximum dose of lg in adults. Use a dose pole to determine dose. Offertetracycline eye ointment for children <6 months	**10%to <30%TF(follicular trachoma) in of children aged 1-9 years:** Treatment of total population in district for at least 3 years **30% TF in children aged 1-9 years:** treatment of total population for a minimum of 5 years **TF of 5-9% in children 1-9 yrs:** targeted treatment based on sub-districts **TF** <5%: S, F, E components only	**Annual.** Must be part of an integrated SAFE strategy (Surgery, Antibiotics, Facial cleanliness, Environmental improvement)
**Onchocerciasis**	Ivermectin 150μ/Kg using dose pole for everyone >5 years (or >90cm), except the chronically ill and pregnant and lactating women during the first week after delivery	**For control:** nodule prevalence >20% or skin microfilaria >40% **For elimination:** under discussion. APOC is using nodule prevalence >5%	**Annual or twice yearly**, (Exceptionally quarterly)
**Lymphatic filariasis**	Albendazole 400 mg for children aged >2 years **plus** diethylcarba-mazine(DEC)6mg/kgin countries where onchocerciasis is not co-endemic, **or** ivermectin 150μ/Kg in countries wehre onchocerciasis is endemic	**Prevalence of >1%**	**Annual.** Treatment must be combined with limb care of patients with elephantiasis or hydrocoele surgery
**Soil-transmitted helminths (STH) {*Ascaris*, hookworms and *Trichuris*)**	Albendazole 400 mg for children above 2 years, or mebendazole 500 mg	**Prevalence >50%:** treat school-aged children, and adults at high risk, twice yearly **Prevalence >20%to <50%:** treat school-aged children once per year. **Prevalence <20%:** individualised treatment. Pre-school children and women of child-bearing age should also be treated (as part of maternal and child health programmes)	**Annual or twice yearly**depending on prevalence. Waterand sanitation strategies must be implemented
**Schistosomiasis**	Praziquantel 40 mg/kg (using dose pole) for children over 4 years (or 94 cms)	**Prevalence >50%:** treat all school-agedchildren. Adults at high risk may also betreated **Prevalence >10%to <50%:** treat childrenonce every two years **Prevalence <10%:** individualised treatment	**Annual treatment** Treatment holidays can be given if prevalence drops. Waterand sanitation strategies must be implemented

Many of the drugs used in MDA can be given together, at one time, so making distribution much more efficient. In Africa, ivermectin should be given with alben-dazole to eliminate LF. Both these drugs have an effect on soil-transmitted helminths (STHs), and ivermectin also kills ectoparasites such as scabies. Where populations are treated for LF, onchocer-ciasis and STHs will be treated at the same time. Praziquantel can also be given with ivermectin and albendazole. At the present time, research is ongoing into co-administration of azithromycin with ivermectin and albendazole, but for the moment there should be an interval of 2 weeks between the administration of azithromycin and the other drugs.

## Precautions

Drugs used in MDA have certain adverse effects and there are several precautions to be taken before using them. Where there is a high worm load in onchocerciasis, there will be symptoms of pain, fever, itching and swelling after treatment, depending on the number of parasites present. These symptoms last for up to 2 days and need symptomatic treatment. Second and subsequent rounds of treatments have far fewer side effects and after three annual treatments there are usually no further adverse effects.

In forested areas of Africa where the parasite *Loa loa* (tropical eye worm) is present, ivermectin should only be given following strict guidelines, otherwise the effects can be severe and sometimes life threatening. Praziquantel should not be given on an empty stomach and will provoke nausea and vomiting in some children, particularly if they have not eaten. Azithromycin also can cause some minor stomach problems. Apartfrom the *Loa loa* situation, the adverse effects are minor and are not contra-indications to treatment; however, in people who suffer with many different parasites, drugs should not all be given together at the first administration.
